# Melatonin inhibits AP-2β/hTERT, NF-κB/COX-2 and Akt/ERK and activates caspase/Cyto C signaling to enhance the antitumor activity of berberine in lung cancer cells

**DOI:** 10.18632/oncotarget.6407

**Published:** 2015-11-27

**Authors:** Jian-Jun Lu, Lingyi Fu, Zhipeng Tang, Changlin Zhang, Lijun Qin, Jingshu Wang, Zhenlong Yu, Dingbo Shi, Xiangsheng Xiao, Fangyun Xie, Wenlin Huang, Wuguo Deng

**Affiliations:** ^1^ Department of Thoracic Surgery, The First Affiliated Hospital, Yat-sen University, Guangzhou, China; ^2^ Sun Yat-sen University Cancer Center, State Key Laboratory of Oncology in South China, Collaborative Innovation Center of Cancer Medicine, Guangzhou, China; ^3^ Guangdong General Hospital, Guangdong Academy of Medical Sciences, Guangzhou, China; ^4^ Institute of Cancer Stem Cell, Dalian Medical University, Dalian, China; ^5^ Department of Pediatrics, Sun Yat-sen Memorial Hospital, Sun Yat-sen University, Guangzhou, China; ^6^ State Key Laboratory of Targeted Drug for Tumors of Guangdong Province, Guangzhou Double Bioproduct Inc., Guangzhou, China

**Keywords:** melantonin, berberine, lung cancer, hTERT, COX-2

## Abstract

Melatonin, a molecule produced throughout the animal and plant kingdoms, and berberine, a plant derived agent, both exhibit antitumor and multiple biological and pharmacological effects, but they have never been combined altogether for the inhibition of human lung cancers. In this study, we investigated the role and underlying mechanisms of melatonin in the regulation of antitumor activity of berberine in lung cancer cells. Treatment with melatonin effectively increased the berberine-mediated inhibitions of cell proliferation, colony formation and cell migration, thereby enhancing the sensitivities of lung cancer cells to berberine. Melatonin also markedly increased apoptosis induced by berberine. Further mechanism study showed that melatonin promoted the cleavage of caspse-9 and PARP, enhanced the inhibition of Bcl2, and triggered the releasing of cytochrome C (Cyto C), thereby increasing the berberine-induced apoptosis. Melatonin also enhanced the berberine-mediated inhibition of telomerase reverses transcriptase (hTERT) by down-regulating the expression of AP-2β and its binding on hTERT promoter. Moreover, melatonin enhanced the berberine-mediated inhibition of cyclooxygenase 2 (COX-2) by inhibiting the nuclear translocation of NF-κB and its binding on COX-2 promoter. Melatonin also increased the berberine-mediated inhibition of the phosphorylated Akt and ERK. Collectively, our results demonstrated that melatonin enhanced the antitumor activity of berberine by activating caspase/Cyto C and inhibiting AP-2β/hTERT, NF-κB/COX-2 and Akt/ERK signaling pathways. Our findings provide new insights in exploring the potential therapeutic strategies and novel targets for lung cancer treatment.

## BACKGROUND

Lung cancer is the leading cause of cancer-related death. The incidence of non-small-cell lung cancer (NSCLC), a major form of lung cancer, has been increasing in the past several decades [1]. Recent advances in lung cancer biology and genetics have suggested many new treatment strategies such as chemotherapy and radiation therapy, however, the outcome is still poor. The management of locally advanced NSCLC has progressed in recent years with the use of combined therapeutic modalities. Thus, optimization of combinations of conventional and novel therapeutic agents may improve the outcome of treatment for lung cancer.

Melatonin (MT) is a phylogenetically well-preserved molecule produced in the pineal gland or derived from plants [2-5]. In addition to its well-known regulatory control of the sleep/wake cycle, as well as circadian rhythms generally, melatonin has a wide range of reported biologic effects, including antioxidant [6-9], free radical scavenging, anti-inflammatory [10-12], and anti-aging activities [13, 14]. There is abundant evidence indicating that melatonin also has important antitumor properties by preventing tumor initiation, promotion, and progression [15-27]. Previous studies have also demonstrated the involvement of the melatonin receptor in mediation of melatonin effects on growth-suppression and gene-modulation in cancer cells [28, 29]. Although evidence of the beneficial effects of melatonin is expanding, the exact molecular mechanisms by which melatonin exerts tumor inhibition effect is unclear, and uncertainty of the combinational treatment of melatonin with other antitumor agents still remains.

Berberine (BBR) is an isoquinoline derivative alkaloid isolated from the rhizome, roots and stem bark of a number of Chinese herbs, the *Berberis* species. It has long history of use for treating diarrhea in traditional Chinese medicine. A growing number of studies reveal that berberine possesses multiple pharmacological activities, including antitumor [30-40], anti-diarrheal [41], anti-hypertensive [42], anti-microbial [43, 44] and anti-inflammatory activities [45-48]. However, so far there has been no investigation concerning the combined treatment of berberine with the natural anticancer agent melatonin for tumor inhibition in human lung cancer.

In this study, we postulated that a combination of melatonin and berberine could achieve the enhanced effects in the inhibition of lung cancer cell growth by targeting multiple cell signaling pathways. To test this possibility, we investigated the combined effects of melatonin and berberine on cell viability, colony formation, cell morphology, cell migration and apoptosis in human NSCLC cells lines H1299 and A549, and further elucidated the underlying mechanisms of actions. Our results showed for the first time that melatonin enhanced the berberine-mediated growth inhibition of lung cancer cells through simultaneous modulation of caspase/cytochrome C, AP-2β/hTERT, NF-κB/COX-2, and Akt/ERK signaling pathways. Our findings provide new insights in exploring the potential therapeutic strategies and novel targets for lung cancer treatment.

## RESULTS

### Melatonin enhanced the berberine-mediated inhibitions of cell proliferation and colony formation

**Figure 1:**
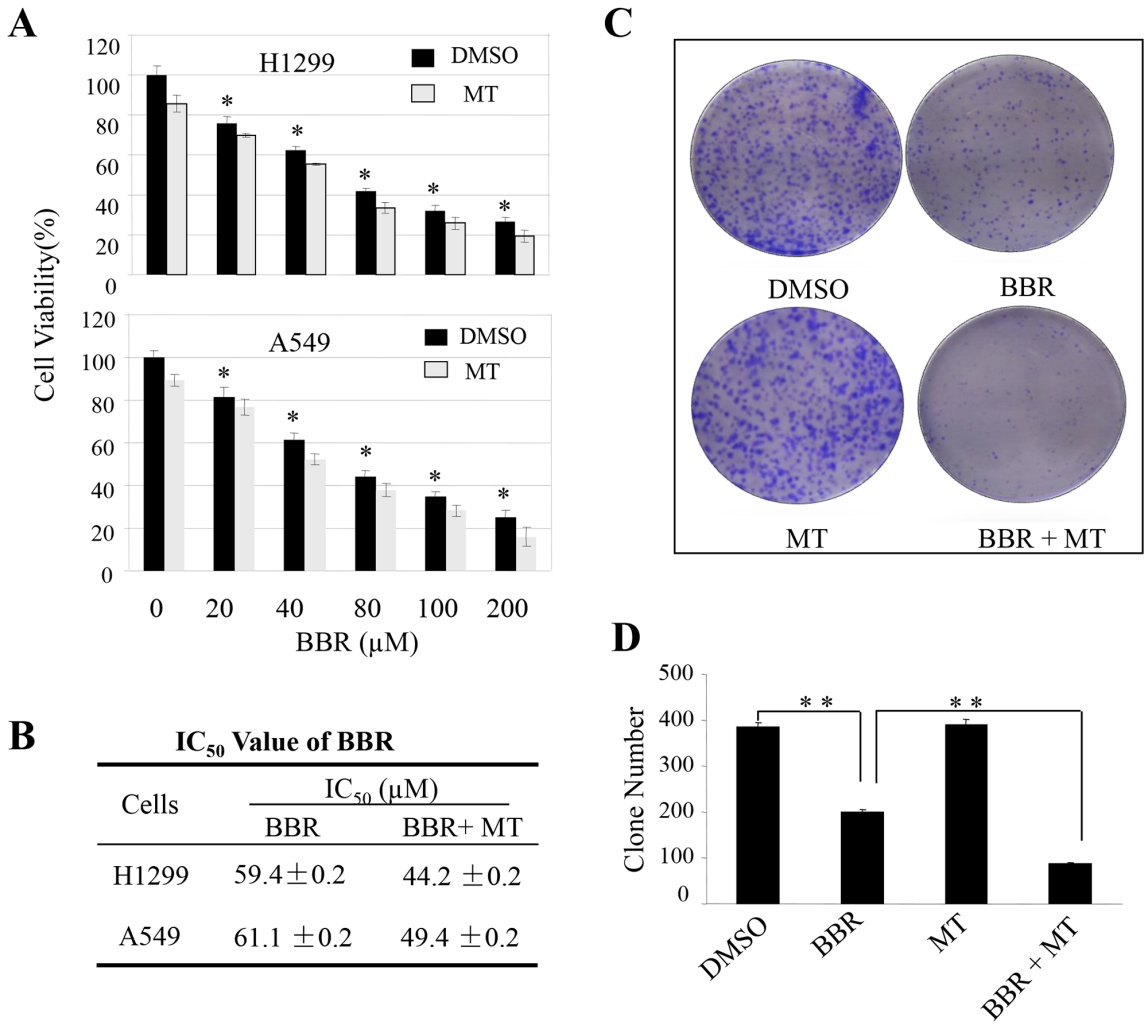
Melatonin enhanced the berberine-mediated inhibitions of cell growth and colony formation **A, B.** Human H1299 and A549 cells were treated with melatonin (MT, 1 mM) and berberine (BBR) at the indicated doses. At 48 hours after treatment, the cell viability (A) was determined by a MTT assay, and the IC_50_ values of BBR for cell viability inhibition (B) in cells treated with or without melatonin were determined. **C, D.** The H1299 cell-induced colony formation was analyzed (C), and the colony formation rate (D) was calculated. Cells treated with DMSO were used as the referent group with cell viability set at 100%. The percent cell viability in each treatment group was calculated relative to cells treated with DMSO vehicle control. The data are presented as mean ± SD of three tests. *P < 0.05, significant differences between treatment groups and DMSO control groups.

We first evaluated the combined effects of melatonin at the pharmacologic concentration (1.0 mM) with berberine at various doses (20 μM to 200 μM) on cell growth inhibitions in H1299 and A549 cells. As shown in Figure 1A, treatment with berberine alone inhibited cell viability in a dose-dependent manner. However, pretreatment of the cells with melatonin markedly enhanced the growth inhibitions of H1299 and A549 cells compared with the treatment with berberine alone (Figure 1A), resulting in a marked reduction of the IC50 values of berberine in inhibiting cell growth (Figure 1B). To confirm the enhanced antitumor activity by melatonin, we also tested the effects of these two agents on tumor cell clonogenicity in H1299 cells. Pretreatment with melatonin (1.0 mM) considerably increased the inhibition of colony formation induced by berberine (100 μM) (Figure 1C), leading to a significant decrease at colony formation ratio by comparison with the treatment with berberine alone (Figure 1D).

### Melatonin enhanced the berberine-mediated cell morphological change and migration inhibition

**Figure 2:**
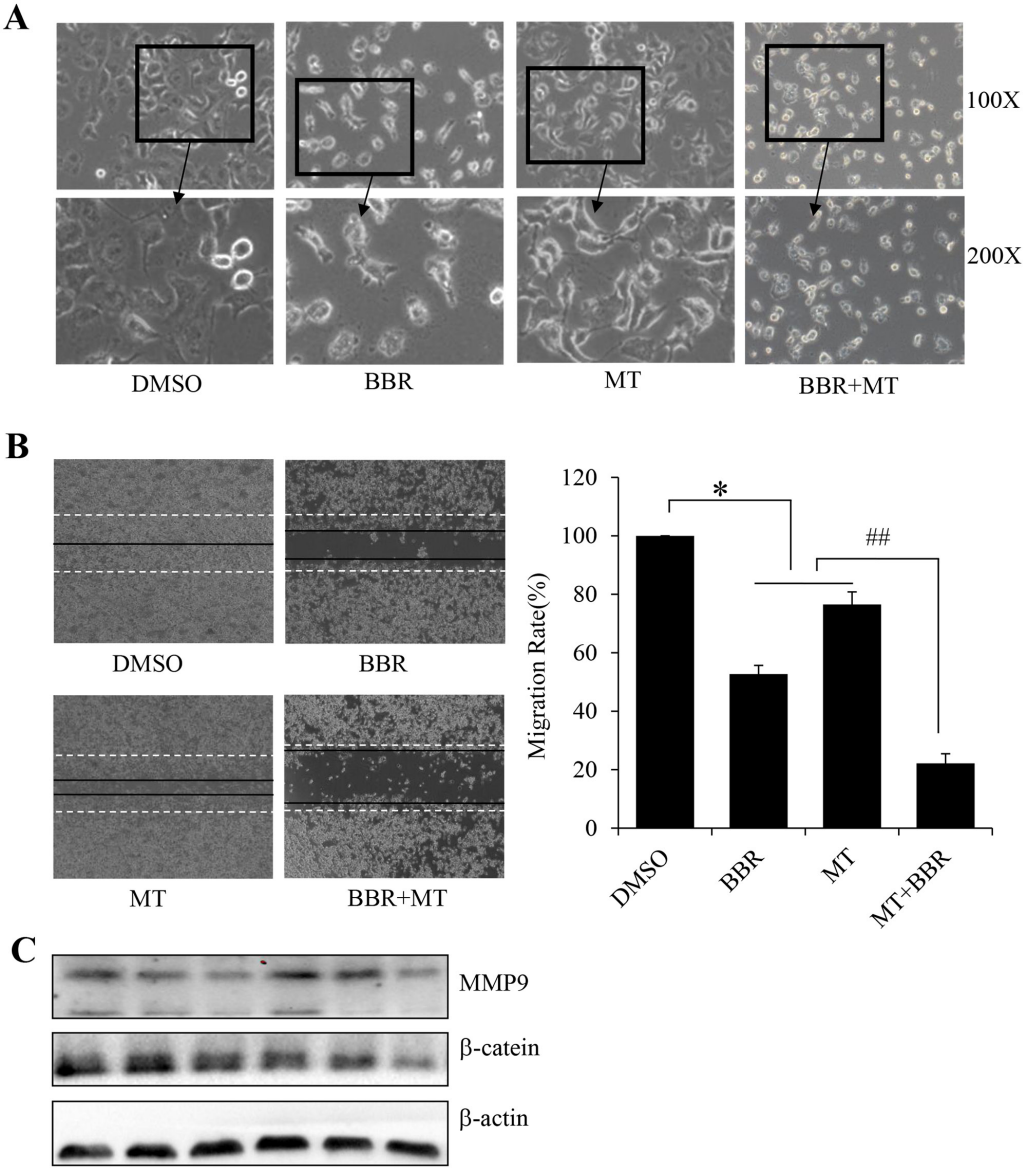
Melatonin enhanced the berberine-mediated cell morphology change and migration inhibition **A.** The changes in cell morphology and spreading in H1299 cells treated with melatonin (MT) (1.0 mM) and berberine (BBR) (100 μM) for 24 h were observed, and cells were photographed using a microscope fitted with digital camera. **B.** Cell migration was analyzed by a scratch assay. H1299 cells were grown to full confluency. The cell monolayers were wounded with a sterile pipette tip, and washed with medium to remove detached cells from the plates. Cells were left either untreated or treated with melatonin (MT) (1.0 mM) and berberine (BBR) (100 μM). After 48 h, the wound gap was observed and cells were photographed. **C.** Protein levels of MMP9 and β-catein were detected after melatonin and berberine treatment. *P < 0.05, significant differences between treatment groups and DMSO control groups. ##*P* < 0.05, significant differences between combination treatment and treatment with melatoninor berberine alone.

We next analyzed the effect of melatonin on the berberine-mediated changes in cell morphology and spreading in H1299 cells. As shown in Figure 2A, the cells treated with melatonin (1.0 mM) or berberine (100 μM) alone formed a cell layer, and more spread and filopodia were observed. By contrast, pretreatment with melatonin markedly enhanced the berberine-induced deduction of cell-to-cell contact and lower spreading with fewer formation of filopodia compared with the treatment with berberine alone, indicating that melatonin promoted the berberine-mediated changes in cell morphology and spreading in NSCLC cells.

We also examined the effect of melatonin on the berberine-mediated inhibition of cell migration in H1299 cells by a wound-healing assay. Consistent with the data from cell growth inhibition and morphology change, pretreatment with melatonin (1.0 mM) considerably increased the erberine-mediated inhibition of cell migration (Figure 2B). The part of gap or wounding space between cell layers after making a scratch was occupied partially by the migrating cells after 48 h in the groups treated with melatonin or berberine alone. However, the empty space of the cells was not occupied by the migrating cells cotreated with melatonin and berberine (Figure 2B). In order to identify the underlying mechanisms involved in cell migration, we also tested the protein levels of MMP9 and β-catein, and found that the protein levels of MMP9 and β-catein were markedly decreased after melatonin and berberine treatment (Figure 2C).

### Melatonin promoted the berberine-induced activation of caspase/cytochrome C dependent apoptosis pathway

**Figure 3:**
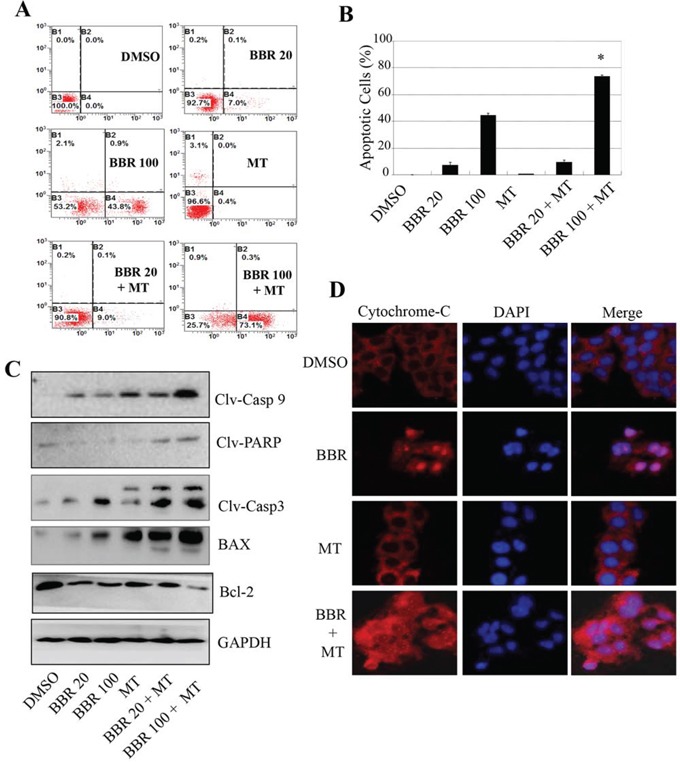
Melatonin enhanced the berberine-induced apoptosis by activating the caspase/cytochrome C signaling **A.** H1299 cells were treated with melatonin (MT, 1.0 mM) and berberine (BBR, 100 μM). At 48 hours after treatment, the apoptosis was determined by a FACS analysis, and the apoptosis from three experiments were calculated **B.** The levels of the cleaved caspase-3, caspase-9, cleaved PARP, BAX and Bcl-2 proteins were analyzed by Western blot **C.** The release of cytochrome-c was analyzed by immunofluorescence imaging analysis to monitor cytochrome-c release from the inter-mitochondrial space into the cytosol **D.** The apoptosis are represented by relative percentages of apoptotic cells versus that in DMSO-treated cells. *P < 0.05, significant differences between the BBR/MT-treated groups and the BBR-treated groups.

We also analyzed the effect of melatonin on the berberine-induced apoptosis in H1299 cells. Treatment with berberine alone at the doses of 20 μM or 100 μM induced 7.1% and 44.7% apoptotic cells (Figure 3A). However, pretreatment with melatonin (1.0 mM) significantly increased the populations of apoptotic cells (Figure 3B), resulting in a 9.1% to 73.4% induction of apoptotic cells.

The activation of caspase cascade forms the essential basis of apoptosis. The release of cytochrome C from the mitochondrial inter-membrane space into the cytosol is the precondition of caspase-dependent apoptosis pathway. Next, we detected the expression of the pro-apoptotic proteins, caspase-9, caspase-3, PARP and BAX, as well as the anti-apoptotic protein Bcl-2, in H1299 cells by Western blot analysis. Pretreatment with melatonin (1.0 mM) effectively enhanced the berberine-mediated up-regulation of the cleaved caspase-3, caspase-9 and PARP as well as BAX and down-regulation of Bcl-2 protein as compared with those treated with berberine alone (Figure 3C), confirming the enhanced effect of melatonin on apoptosis induction.

We also performed immunofluorescence imaging (IFI) analysis to monitor the changes in the subcellular localization of cytochrome C in H1299 cells to examine whether the melatonin could enhance the berberine-induced cytochrome C releasing. Treatment with berberine (100 μM) alone also induced the release of cytochrome C from the inter-mitochondrial space into the cytosol. However, pretreatment with melatonin (1 mM) effectively promoted the releasing of cytochrome C compared to the treatment with berberine alone (Figure 3D). These results demonstrate that melatonin may facilitate the downstream cytochrome C dependent apoptosome assembly and caspase activation in the cytosol in lung cancer cells.

### Melatonin enhanced the berberine-mediated suppression of the AP-2β/hTERT signaling

**Figure 4:**
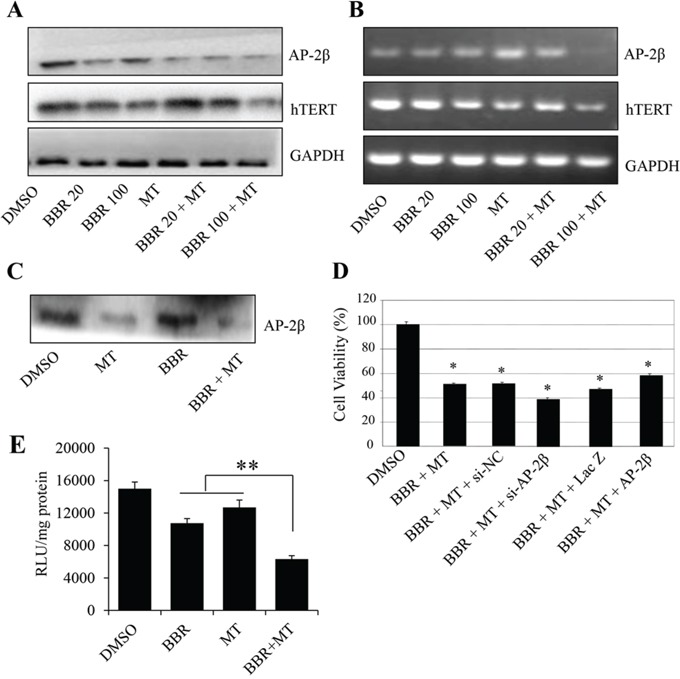
Melatonin enhanced the berberine-mediated inhibition of AP-2β/hTERT signaling **A, B.** Human H1299 cells were treated with melatonin (MT, 1.0 mM) or berberine (BBR, 20 μM and 100 μM). At 48 hours after treatment, the AP-2β and hTERT proteins (A) and mRNA (B) were analyzed by Western blotting and RT-PCR, respectively. GAPDH were used as controls for sample loading. **C.** H1299 cells were treated with melatonin (MT, 1.0 mM) or berberine (BBR, 100 μM). At 48 hours after treatment, the binding of AP-2β to hTERT promoter probe was analyzed by a streptavidin-agarose pulldown assay. **D.** H1299 cells were transfected with an AP-2β siRNA or an AP-2β-expressing vector for 24 hours, and then treated with melatonin (MT, 1.0 mM, and berberine (BBR, 100 μM). At 48 hours after treatment, cell viability was determined by a MTT assay. The percent cell viability in each treatment group was calculated relative to cells treated with the vehicle control. **E.** H1299 cells were treated with melatonin (MT, 1.0 mM) and berberine (BBR, 100 μM) after transfection with hTERT promoter-driven luciferase plasmids. The proteins were extracted, and luciferase activity was detected by luciferase reporter assay kit. The data are presented as the mean ± SD of three separate experiments. *P < 0.05, significant differences between treatment groups and DMSO control groups.

hTERT is a hallmark of lung tumorigenesis. It is highly expressed in lung cancer cells and tightly regulated by transcriptional factor AP-2β [49]. To determine whether melatonin also affect the AP-2/hTERT signaling in NSCLC cells, we treated NSCLC cells with melatonin (1.0 mM) or berberine (20 μM or 100 μM) alone or altogether, and examined expression of AP-2β and hTERT at protein and mRNA levels by Western blot and RT-PCR, respectively. Treatment with berberine alone downregulated the expression of hTERT and AP-2β at protein (Figure 4A) and mRNA levels (Figure 4B). However, pretreatment with melatonin (1 mM) considerably enhanced the berberine-mediated inhibitions on AP-2β and hTERT proteins (Figure 4A) and mRNAs (Figure 4B) levels by comparison with those treated with either berberine alone.

Since hTERT expression is tightly controlled by the binding activity of AP-2β on hTERT promoter, we next performed streptavidin-agarose pulldown assay to examine the effect of melatonin on AP-2β binding activity in H1299 cells. Treatment with berberine and melatonin considerably suppressed the expression of AP-2β protein (Figure 4A), thereby inhibiting the binding of AP-2β to the hTERT promoter (Figure 4C). In addition, we also detected the effects of berberine and melatonin on hTERT promoter activity. The results showed that pretreatment with melatonin effectively enhanced the berberine-mediated inhibition on hTERT promoter activity (Figure 4E).

To further confirm that the AP-2β signaling is involved in the melatonin-mediated enhancement of inhibition of cell growth, H1299 cells were transfected with an AP-2β specific siRNA (100 nM) or an AP-2β-expressing vector and then cotreated with melatonin (1.0 mM) and berberine (100 μM). As shown in Figure 4D, by comparison with the non-specific siRNA control (si-NC), knockdown of AP-2β expression by AP-2β siRNA (si-AP-2β) slightly increased the cell growth inhibition mediated by melatonin and berberine. Conversely, overexpression of AP-2β by transfection the expressing vector (AP-2β) effectively reversed the inhibition of cell growth as compared with the transfection with the control vector LacZ (Figure 4D). These results show that the enhancement of growth inhibition by melatonin is mediated at least in part through inhibition of AP-2β/hTERT signaling pathway in NSCLC cells.

### Melatonin enhanced the berberine-mediated inhibition of the NF-κB/COX-2 signaling

**Figure 5:**
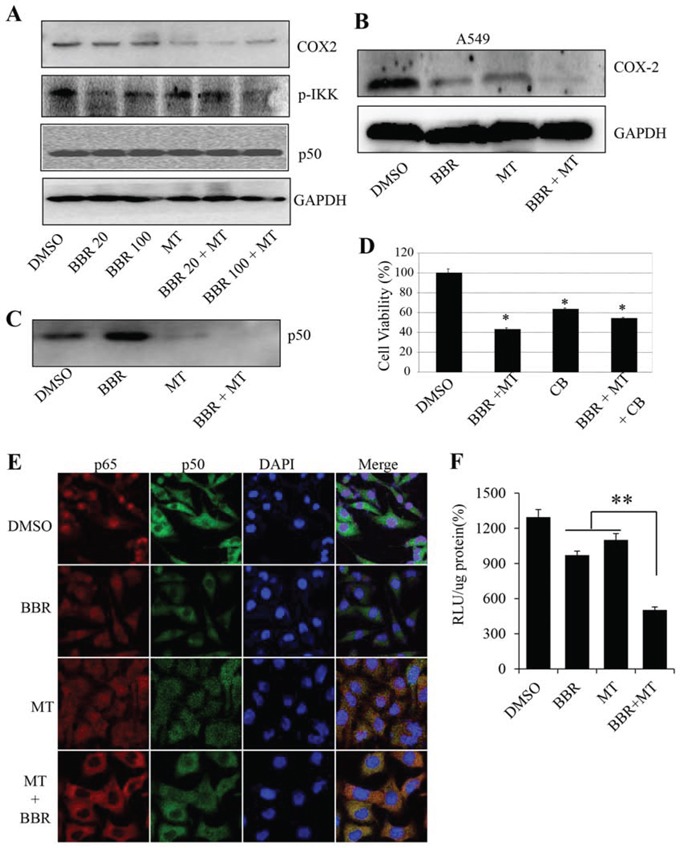
Melatonin enhanced the berberine-mediated inhibition of p50/COX-2 signaling **A.** Human H1299 cells were treated with melatonin (MT, 1.0 mM) and berberine (BBR, 20 μM and 100 μM). At 48 hours after treatment, the p-IKK, COX-2 and p50 proteins (A) were analyzed by Western blotting. GAPDH were used as controls for sample loading. **B.** Human A549 cells were treated with melatonin (MT, 1.0 mM) and berberine (BBR, 50 μM). At 48 hours after treatment, the COX-2 protein was analyzed by Western blotting. **C.** H1299 cells were pretreated with the COX-2 selective inhibitor celecoxib (CB) (20 μM) for 24 hours, and then treated with melatonin (MT, 1.0 mM) and berberine (BBR, 100 μM). At 48 hours after treatment, cell viability was determined by MTT analysis. The percent cell viability in each treatment group was calculated relative to cells treated with the vehicle control. **D.** H1299 cells were treated with melatonin (MT, 1.0 mM) and berberine (BBR, 100 μM). At 48 hours after treatment, the binding of p50 to COX-2 promoter probe was analyzed by a streptavidin-agarose pulldown assay. **E.** H1299 cells grown on chamber slides were treated with melatonin melatonin (MT, 1.0 mM) and berberine (BBR, 100 μM). At 48 hours after treatment, the subcellular localization of p50 and p65 was examined by a confocal microscope. More than 100 cells were inspected per experiment, and cells with typical morphology were presented. **F.** H1299 cells were treated with melatonin (MT, 1.0 mM) and berberine (BBR, 100 μM) after transfection with COX-2 promoter-driven luciferase plasmids. The luciferase activity was detected by luciferase reporter assay kit. The data are presented as the mean ± SD of three separate experiments. *P < 0.05, significant differences between treatment groups and DMSO control groups.

The COX-2 signaling is closely implicated in lung cancer cell growth, migration and angiogenesis [50, 51]. To determine the effect of melatonin on COX-2 signaling in NSCLC cancer cells, we analyzed the expression of COX-2 protein in the H1299 and A549 cells treated with melatonin or berberine alone or altogether by Western blotting. Treatment with berberine alone did not significantly inhibited COX-2 protein expression. However, pretreatment of melatonin (1.0 mM) in NSCLC cells markedly increased the inhibition of COX-2 protein expression in the berberine-treated H1299 and A549 cells (Figure 5A and 5B).

To confirm that melatonin enhanced the inhibition of COX-2 signaling, H1299 cells were treated with a COX-2-selective inhibitor celecoxib (20 μM), and followed the treatment of melatonin (1.0 mM) and berberine (100 μM). As shown in Figure 5D, pretreatment with celecoxib (CB) alone also inhibited cell viability, whereas a combined treatment with melatonin and berberine did not significantly alter cell viability inhibition mediated by the COX-2-selective inhibitor, indicating that COX-2 signaling is also an important target for melatonin in NSCLC cells.

The expression of COX-2 is tightly regulated by the binding activity of p50 NF-κB on COX-2 promoter structure. We next determined whether melatonin also inhibited the binding of p50 NF-κB on COX-2 promoter in H1299 cells. Streptavidin-agarose pulldown assay showed that treatment with berberine did not change the binding activity of p50 NF-κB on COX-2 promoter, however, pretreatment with melatonin markedly inhibited p50 NF-κB binding to COX-2 promoter compared with those treated with berberine alone (Figure 5C). The expression of p50 protein was not changed by melatonin and berberine alone or altogether (Figure 5A). Moreover, we also detected the effect of the combined treatment on COX-2 promoter activity. The results showed that pretreatment with melatonin effectively enhanced the berberine-mediated inhibition of COX-2 promoter activity (Figure 5F).

The translocation of NF-κB in cell nuclei and cytoplasm plays a key role in regulating COX-2 gene expression. We next performed immunofluorescence assay to evaluate the effect of melatonin on p50 and p65 NF-κB translocation in H1299 cells by a confocal microscope. Constitutive translocation of NF-κB p50 and p65 to the cell nuclei was detected (Figure 5E). Treatment with berberine (100 μM) alone also caused slight translocation of the p50 and p65 from cell nuclei to cytoplasm. However, pretreatment with melatonin effectively enhanced the berberine-mediated nuclear translocation of p50 and p65 NF-κB. The results indicate that the enhanced inhibition of tumor cell growth by melatonin might be also mediated partially via the p50/p65 NF-κB/COX-2 signaling pathway in NSCLC cells.

As melatonin and berberine regulated the translocation of NF-kb in cell nuclei and cytoplasm, next we detected the effect of melatonin and berberine on the up-stream regulator of NF-kB, phosphorylation of IKK. As shown in Figure 5A, treatment with melatonin and berberine alone or combination both could decrease the level of p-IKK, and pretreatment with melatonin effectively enhanced the berberine-mediated decrease on p-IKK.

### Melatonin enhanced the berberine-mediated inhibition of the Akt/ERK signaling

**Figure 6:**
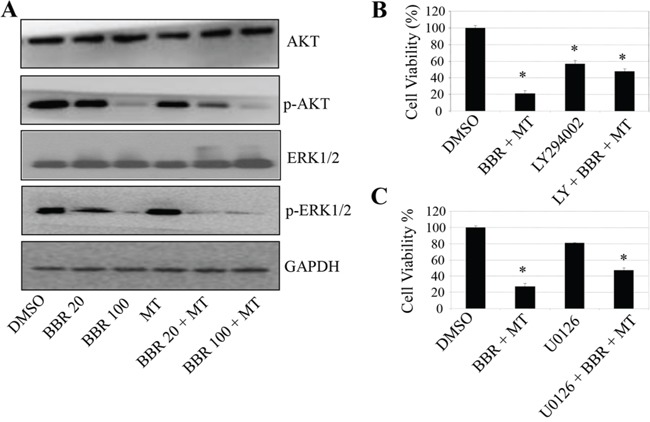
Melatonin enhanced the berberine-mediated inactivation of Akt/ERK signaling **A.** H1299 cells were treated with melatonin (MT, 1.0 mM) and berberine (BBR, 20 or 100 μM). At 48 hours after treatment, the expression of the total and phosphorylated Akt and ERK1/2 proteins were determined by Western blot. **B, C.** H1299 cells were treated with the Akt-selective inhibitor LY294002 (LY, 30 μM) (B) or the Mek1/2-selective inhibitor U0126 (50 μM) (C) for 24 hours, respectively, and then treated with melatonin (MT, 1.0 mM) and berberine (BBR, 100 μM). At 48 hours after treatment, cell viability was determined by MTT analysis. The percent cell viability in each treatment group was calculated relative to cells treated with the vehicle control. The data are presented as the mean ± SD of three separate experiments. *P < 0.05, significant differences between treatment groups and control groups.

The PI3K/Akt and Raf/MEK/ERK signaling play important roles in tumor cell growth and is implicated in the expression of the cancer-related genes such as hTERT and COX-2. To determine whether the melatonin-mediated enhancement of cell growth inhibition is also through the inhibitions of the PI3K/Akt and Raf/MEK/ERK signaling pathway, we next analyzed the effect of melatonin on Akt and ERK phosphorylation in the berberine-treated H1299 cells by Western blot. As shown in Figure 6A, treatment with berberine (20 μM and 100 μM) inhibited the phosphorylation of Akt and ERK1/2 proteins, whereas pretreatment with melatonin (1.0 mM) effectively increased the berberine-mediated inhibition of the phosphorylation of Akt and ERK1/2 proteins. The total protein levels of Akt and ERK1/2 did not change by melatonin and berberine alone or altogether.

To further confirm that melatonin targets Akt/ERK signaling to inhibit NSCLC cell growth, we next analyzed the effect of an Akt or ERK-selective inhibitor on the melatonin and berberine-mediated inhibition of cell viability in H1299 cells. Treatment with LY29400, the Akt inhibitor or U0126, the inhibitor of Mek1/2 (the direct upstream molecule of ERK1/2) effectively inhibited cell viability. However, pretreatment with these inhibitors did not significantly change the cell growth inhibitions mediated by melatonin (Figure 6B), indicating that the Ak/ERK signaling is an important target for melatonin in enhancing the berberine-mediated growth inhibition in NSCLC cells.

### Melatonin enhanced the berberine-mediated inhibition of lung cancer growth in a xenograft mouse model *in vivo*

**Figure 7:**
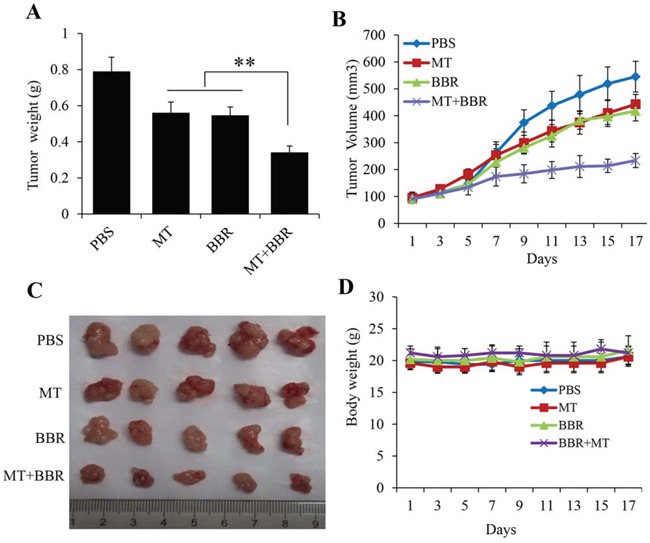
Melatonin enhanced the berberine-mediated inhibition of lung cancer growth in a xenograft tumor model *in vivo* A549 cells were injected subcutaneously into the nude mice to evaluate the effects of MT and BBR. **A.** Tumor weight, **B.** Tumor volumes at different time, **C.** Tumor pictures. **D.** Body weights of the mice, *P < 0.05, significant differences between treatment groups and control groups. Five mice number for each group.

To confirm the enhanced berberine-mediated inhibition of lung cancer growth by melatonin, we analyzed the effects of melatonin and berberine treatment on tumorigenicity *in vivo* using a A549 xenograft mouse model. After administration with MT and BBR alone or together for 17 days, both the tumor weights (Figure 7A) and tumor volumes (Figure 7B, 7C) were inhibited by treating with MT and BBR alone, however, treatment with MT and BBR together markedly enhanced the growth inhibitions of xenograft compared with the treatment with BBR alone. The combined treatment did not affect significantly the body weight weights of the mice (Figure 7D). These results supported that MT could enhance BBR-mediated inhibition of the xenografted human lung cancer cell's growth without the remarkable adverse effects.

## DISCUSSION

In this study, we investigated the response of human lung cancer cells to the combined treatment of melatonin and berberine. Melatonin effectively enhanced the berberine-mediated cell growth inhibition and apoptosis induction in NSCLC cells. Our results also showed that the enhancement of tumor cell growth inhibition by melatonin is mediated through simultaneous modulation of caspase/cytochrome C, AP-2β/hTERT, NF-κB/COX-2, and Akt/ERK dependent signaling pathways. To the best of our knowledge, it might be the first time to report that melatonin sensitized human lung cancer cells to berberine and to demonstrate the underlying mechanisms of action.

Melatonin plays an important role not only in the regulation of the circadian rhythm but also in the modulation of cancer risk by acting as an anti-inflammatory agent and an antioxidant. Melatonin has been shown to potentiate flavone-induced apoptosis in human cancer cells by increasing the level of glycolytic end products [52]. It also helps decrease angiogenesis in cancer cells, which means that it helped block blood supply to the tumor, resulting in tumor suppression. Melatonin is capable of modulating the signaling pathways associated with anti-proliferation and pro-apoptotic effect in cancer cells to inhibit growth of different tumors. Berberine has been shown to exhibit a broad range of pharmacological properties such as anti-tumor, anti-angiogenesis, and anti-metastasis activities. Both melatonin and berberine have respectively been shown to inhibit cancer cell growth in a huge number of studies, and they have been used in combination with other chemotherapeutic agents in various cancer cells, but they have never been combined altogether as an anti-lung cancer treatment. In this study, we hypothesized that melatonin might play a role in sensitizing or synergizing lung cancer cellular response to berberine treatment and actually analyzed the combined effect of melatonin and berberine on cell proliferation, migration, and apoptosis in lung cancer cells. We found that melatonin indeed potentiated the effects of berberine alone on cell growth inhibition and apoptosis induction. All the results might serve as a basis for guiding the combinational treatment of natural anticancer compounds in improving therapy efficiency for lung cancer. However, the combination of melantonin and berberine made an additive but not a synergistic anti-tumor effect. Therefore, further investigations about the clinical significance of this combination therapy are needed.

The activation of caspase cascade forms the essential basis of apoptosis. The release of cytochrome C from the mitochondrial inter-membrane space into the cytosol is the precondition of caspase-dependent apoptosis pathway. In this study, we have also shown that melatonin markedly enhanced activation of caspases and promoted releasing of cytochrome C from mitochondria to cytosol.

hTERT is an important component of human telomerase, which lengthens chromosome ends and maintains chromosomal stability, leading to cellular immortalization [53]. It is commonly overexpressed in a wide range of human cancers, including lung cancer [54]. Inhibition of hTERT expression was found to contribute to prevent proliferation and angiogenesis and to induce apoptosis of human cells. hTERT expression is transcriptionally controlled by the binding of activating enhancer-binding protein-2β (AP-2β) to the corresponding sites located in their promoters [49]. By binding to the hTERT promoter, AP-2β exerts its biological effects through activation of the tumor-related gene hTERT. AP-2β factors orchestrate a variety of cell processes including apoptosis, cell growth, and tissue differentiation during embryogenesis. Gene knockout experiments with AP-2β have shown that AP2-β-deficient mice die shortly after birth owing to collecting duct and distal tubular epithelial defects caused by massive renal epithelial cell apoptosis. However, no information is available about the regulation of AP-2β/hTERT signaling by melatonin and berberine in human lung cancer cells. In our study, we showed that melantonin down-regulated the expression of AP-2β and hTERT, thereby inhibited cell proliferation. When AP-2β was increased by overexpression with the use of melantonin, the melatonin-mediated cell growth inhibition was rescued. However, further study about the expression of hTERT, the target gene of AP-2β, is needed in the rescue experiment.

COX-2 overexpression commonly appears in a wide range of human cancers, including lung cancer. Its expression and the sequential PGE2 production could upregulate EGFR, PI3K, and ERK1/2 signaling to induce angiogenesis, cell proliferation, invasion, and metastasis of tumor cells. Melatonin has been shown to play anti-tumor and anti-inflammation roles partially through inhibiting COX-2 expression [55]. Our current study also demonstrated that melatonin enhanced berberine-mediated inhibition of COX-2 expression in NSCLC cells.

COX-2 expression is transcriptionally controlled by the binding of multiple transactivators and coactivators to the corresponding sites located in its promoter. Among the known several regulatory elements distributing in the core promoter region of COX-2 transcription start site, NF-κB binding site is essential for COX-2 promoter activity [56, 57]. Because melatonin enhanced the inhibition of COX-2 expression, we were interested in whether melatonin would modulate NF-κB signaling in NSCLC cells. In our study, we confirmed the nuclear localization of p50 NF-κB in NSCLC cells. We found that the enhanced inhibition of COX-2 expression by melatonin is partially mediated by stimulating p50 NF-κB translocation from cell nuclear to cytosol. We also further demonstrated the increased inhibitory effects of melatonin in NSCLC cells were mediated by inhibiting the binding of p50 NF-κB to COX-2 promoter. Further studies are needed to elucidate the mechanisms by which melatonin enhances the inhibitions of p50 acetylation and phosphorylation.

**Figure 8:**
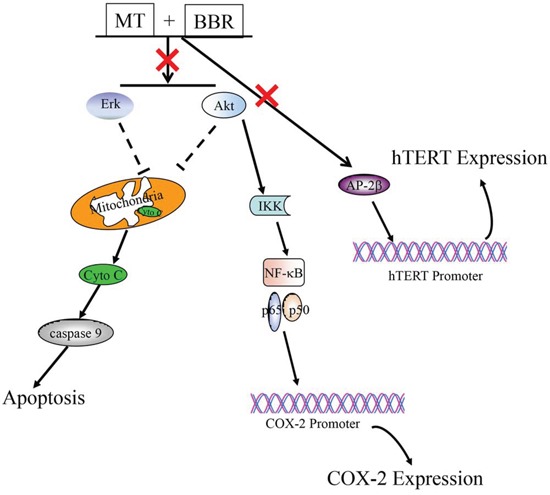
The schematic diagram regarding the regulation of multiple signaling pathways by melatonin and berberine in lung cancer cells

In summary, melatonin sensitized NSCLC cells to berberine and enhanced the growth inhibitory effect of berberine by simultaneously targeting caspase/cytochrome C, AP-2β/hTERT, NF-kB/COX-2, Akt/ERK signaling pathways (Figure 8). These findings provide new insights into understanding the molecular mechanisms by which melatonin sensitizes NSCLC cells to berberine treatment, and suggest some clues for the development of new therapeutic strategies in human lung cancer therapy. Further studies are warranted to more firmly establish this supposition.

## MATERIALS AND METHODS

### Cell culture

The human lung cell lines H1299 and A549 were obtained from American Type Culture Collection (ATCC, Manassas, VA). Cells were cultured as monolayers in RPMI 1640 culture media supplemented with 10% heat-inactivated fetal bovine serum, 100 μg/ml penicillin and 100 μg/ml streptomycin, and maintained in an incubator with a humidified atmosphere of 95% air and 5% CO2 at 37°C. The H1299 cells were treated with 100 nm PMA for 8 hours.

### Reagents and antibodies

Melatonin, berberine and celecoxib were purchased from Sigma (St. Louis, MO) and dissolved in a small amount of DMSO before addition to the complete cell culture medium. Streptavidin-agarose was purchased from Sigma (St. Louis, MO). Antibodies to GAPDH, AP-2β, hTERT, COX-2, p50, cytochrome-c, PARP, caspase-9, BAX and Bcl-2 were obtained from Santa Cruz Biotechnology (Santa Cruz, CA). Antibodies against Akt, ERK1/2, phosphrylated Akt or ERK1/2 were purchased from Cell Signaling (Beverly, MA).

### Cell viability assay

Cell viability was determined using MTT assay (Roche Diagnosis, Indianapolis, IN). Briefly, lung cancer cell lines were seeded at 4 × 10^3^ cells/well in 96-well plates. Cells were cultured for overnight, and then the cells were changed to fresh medium containing various concentrations of melatonin and berberine dissolved in DMSO (final concentration, 0.1%). After the cells were incubated for 48 h, the growth of cells was measured. The effect on cell viability was assessed as the percent cell viability compared with vehicle-treated control cells, which were arbitrarily assigned 100% viability. The berberine concentration required to cause 50% cell growth inhibition (EC50) was determined by interpolation from dose-response curves.

### Anchorage independent colony formation

Cells plated in 6-well plates were treated with melatonin or berberine alone or altogether. After 24 h, cells were washed with PBS and trypsinized. Then cells (8 × 10^3^/ml) were mixed in 1 ml of 0.3% McCoy's 5a agar containing 10% FBS. The cultures were maintained in a 37°C, 5% CO_2_ incubator for 21 days. The medium was discarded and the cells were carefully washed with PBS twice. After being fixed with 4% paraformaldehyde for 15 min, the cells were stained with 0.1% crystal violet for 15 min before washing with tap water and air-drying. The colonies with more than 50 cells were counted with an ordinary optical microscope. The colony formation rate was calculated with the following formula: Plate colony formation inhibitory ratio = (number of colonies treated with melatonin or berberine/number of cells inoculated) × 100%.

### Wound-healing assay

Wound-healing assay was performed to detect cell migration. The cells were grown to full confluency in six-well plates and incubated overnight in starvation medium. Cell monolayers were wounded with a sterile 100 μL pipette tip, washed with starvation medium to remove detached cells from the plates. Cells were treated with indicated doses of melatonin or berberine in full medium and kept in a CO2 incubator. After 48 h, medium was replaced with PBS, the wound gap was observed and cells were photographed using an Olympus microscope fitted with digital camera.

### Apoptosis assay

Apoptosis were measured by flow cytometry using Annexin-V staining-based fluorescence-activated cell sorter (FACS). In brief, cells plated in 6-well plates were treated with melatonin or berberine alone or altogether. At 48 h after treatment, cells were collected and washed once with cold PBS, and subsequently stained with Annexin V (Invitrogen, Carlsbad, CA). Stained cells were analyzed by flow cytometry.

### Western blot analysis

Cell lysate proteins were separated by electrophoresis in a 10% sodium dodecyl suplfate-polyacrylamide minigel (SDS-PAGE) and electrophoretically transferred to a PVDF membrane. Western blots were probed with the specific antibodies. The protein bands were detected by enhanced chemiluminescence.

### Reverse transcription-polymerase chain reaction (RT-PCR)

Total cellular RNA was extracted with Tri-Zol reagent (Life Technologies, Glasgow, UK) according to the manufacturer's instructions. Total RNA was reverse-transcribed by using the Superscript™-III kit (Invitrogen, Carlsbad, CA). PCR analysis was performed on aliquots of the cDNA preparations to detect gene expression. The amplified products were visualized on 1% agarose gels. PCR conditions were 4 min at 94°C followed by 30 cycles (25 for GAPDH): 30 seconds at 94°C, 30 seconds at 60°C, and 1 min at 72°C,

### Transfection

The transfection of siRNAs or expressing vectors were performed by Lipofectamine 2000 reagent according to the manufacturer's protocol (Invitrogen, Carlsbad, CA).

### DNA-protein binding by streptavidin-agarose pulldown assay

Binding of AP-2β or p50 NF-κB to hTERT or COX-2 core promoter probes were determined by a streptavidin-agarose pulldown assay. A biotin-labeled double-stranded probe corresponding to hTERT or COX-2 promoter sequence was synthesized. The binding assay was performed by mixing 400 μg of nuclear extract proteins, 4 μg of the biotinylated DNA probe and 40 μl of 4% streptavidin-conjugated agarose beads at room temperature for 1 h in a rotating shaker. Beads were pelleted by centrifugation to pull down the DNA-protein complex. After washing, proteins in the complex were analyzed by immunoblotting using antibodies (1 μg/ml each) specific for AP-2β or p50 NF-κB. The mixture was incubated at room temperature for 1 h with shaking, and centrifuged to pull down the DNA-protein complex. DNA-bound AP-2β or p50 NF-κB protein was dissociated and analyzed by Western blotting. A non-immune rabbit IgG (1 μg/ml) was also used as negative controls.

### Confocal immunofluorescence

For confocal microscopy analysis, cells grown on chamber slides were washed in PBS and fixed for 15 min at room temperature with 4% paraformaldehyde. The samples were pretreated with 10% bovine serum albumin (BSA) in PBS for 30 min. Antibodies against p50 in the blocking solution were added to the sample and incubated for overnight at 4°C. Nonimmune IgG was included as controls. Following five 5-min washes with PBS, fluorescein isothiocyanate- and rhodamineconjugated secondary antibodies were added in blocking solutions and incubated for 1 hour. After five additional 5-min washes, samples were examined with a Zeiss LSM700 confocal microscope, and images were processed with Zen2010 software. More than 100 cells were inspected per experiment, and cells with typical morphology were presented.

### Animal study

The animals used in the present study were BALB/c nude male mice (4 weeks old), which were purchased from Dalian medical university SPF Laboratory Animal Center (Dalian, China). The nude mice, weighing between 18 and 20 g, were fed under specific pathogen-free conditions. All procedures were in accordance with the National Institutes of Health Guide for the Care and Use of Laboratory Animals (National Institutes of Health, Bethesda, MD, USA). To evaluate the therapeutic efficacy of MT and BBR in a human A549 orthotopic lung cancer mouse model, A549 cells (5 × 10^6^ in 100 μL PBS) were injected subcutaneously near the axillary fossa of the nude mice. Two weeks later, when the tumor diameters reached 3 mm × 4 mm, the tumor cell-inoculated mice were randomly divided into four treatment groups that each contained five mice, group A treated with PBS; group B with 25 mg/kg MT; group C with 10 mg/kg BBR; group D with MT and BBR by intraperitoneal injection every day. Tumors were measured with a caliper every 2 days, and the tumor volume was calculated using the formula V = 1/2 (width^2^ × length). Body weights were also recorded. On day 30 after tumor cell inoculation, all experimental mice were terminated with ether anesthesia and the total weight of the tumors in each mouse was measured.

### Statistical analysis

Analysis of variance and Student's t test were used to compare the values of the test and control samples. *P* < 0.05 was considered to a statistically significant difference. SPSS6.0 software was used for all statistical analysis. The significance was analyzed by the paired t test. All the experiments were done three times, and mean values and standard deviation were calculated.
